# Combined gastric mucosal ablation of the fundus using a new hybrid device with endoscopic sleeve gastroplasty for obesity

**DOI:** 10.1055/a-2767-0830

**Published:** 2026-01-28

**Authors:** Ivo Boškoski, Loredana Gualtieri, Martina De Siena, Maria Valeria Matteo, Valerio Pontecorvi, Cristiano Spada, Vincenzo Bove

**Affiliations:** 1 18654 Digestive Endoscopy Unit, Fondazione Policlinico Universitario Agostino Gemelli IRCCS, Rome, Italy

Endoscopic bariatric therapies provide a minimally invasive alternative for patients with obesity who are either ineligible for or unwilling to undergo surgery 1 2 .

We describe a novel combined endoscopic approach that integrates endoscopic sleeve gastroplasty (ESG) with gastric mucosal ablation (GMA) of the fundus using a MOVIVA probe (ERBE, Tübingen, Germany).

The MOVIVA probe integrates two essential functions. First, it enables the injection of a submucosal fluid cushion to lift the mucosa and shield the deeper tissue layers from potential thermal damage. Second, it allows controlled superficial ablation of the mucosa through argon plasma coagulation. The goal is to reduce ghrelin-producing cells, thereby attenuating hunger signaling. Obese patients show increased ghrelin activity, which is correlated with the body mass index (BMI 3 ). Targeting fundic ghrelin secretion has been associated with approximately 10% of the total body weight loss at 12 months 4 5 .

A 54-year-old male patient, with a BMI of 44 kg/m ^2^ , type 2 diabetes mellitus, arterial hypertension, and obstructive sleep apnea syndrome, was selected after multidisciplinary evaluation.

The procedure was performed under general anesthesia in a supine position using a single-channel gastroscope. The MOVIVA probe was first introduced to achieve targeted mucosal ablation of the gastric fundus, providing uniform surface coverage. Subsequently, ESG was performed with an OverStitch NXT suturing system (Boston Scientific, Massachusetts, USA). Full-thickness U-shaped sutures were placed along the greater curvature to reduce gastric lumen ( Video 1 ).

The video illustrates the complete case report, detailing the endoscopic technique and the specific hybrid devices used during the procedure, highlighting its feasibility and clinical applicability.Video 1

The procedure was uneventful. Oral intake was resumed the following day. At the 1-month follow-up, the patient reported marked early satiety and achieved 16.5% of total body weight loss. No adverse events were reported.

This novel approach combines mechanical restriction with hormonal modulation. ESG remodels the gastric lumen, while MOVIVA-assisted GMA augments the metabolic effect by targeting ghrelin secretion. Future studies are needed to validate long-term safety and efficacy of this combined therapy.

Endoscopy_UCTN_Code_TTT_1AO_2AN
